# Some Observations on Cholera

**Published:** 1832-07

**Authors:** T. Ridgway


### CHOLERA.

*Some Observations on Cholera.* By T. Ridgway, M.D.

It is perhaps remarkable, that at Dover, situated as it is in the direct line of communication between the two great capitals of London and Paris, and in hourly intercourse with Calais, in no instance has cholera, in its malignant shape, yet appeared. Here, however, among the persons that come under my care, who are youthful, robust, well fed, and well clothed, the disease, in its milder modifications, has existed from the middle of the last summer; and, as I have every reason to believe, the same thing has happened among the general mass of the inhabitants.

About the 10th of August, after a short interval which succeeded to a considerable prevalence of summer catarrh, the first cases of cholera (coming several of them together,) at once presented themselves. They did not persist, but from the beginning became less frequent, recurring singly, and at intervals, throughout the following month of September, and then gradually subsiding, until, in October, they seemed to have disappeared altogether, being followed by, or resolving themselves into the common form of continued fever. But in November other cases arose, the designation of which, because of the absence of vomiting, and from a closer observation of the disease, was changed into Erythematic Enteritis; a term derived from medical writers, and the adoption whereof will serve to shew the opinion entertained of the seat and nature of the affection.

In like manner with the first, these also soon subsided, but continuing to recur at intervals to the present time; sometimes seeming to disappear, and again threatening to revive, and being interspersed, although rarely, with cases resembling the former, whereof they seem to be but a modified continuation, and into which they appear the more and more disposed to return as the spring advances.

In their course these affections have been accompanied by a few slight cases of continued fever, which often appear but as the imperfect developements of disease; and, on the whole, they have seemed to supersede the more important forms of acute disease, as far as my observation extends, nearly altogether.

The earlier cases of this cholera were benign, as they have shewn themselves throughout: but they appeared to me to exhibit peculiarities which, at first view, served to distinguish them, in my mind, from such as I have seen on former occasions. They came together in unusual number; the general disturbance seemed less remarkable, the local affection more definite and fixed; the evacuations were less frequent and less free, with accumulation in the intestinal cavity; and there was a disposition to protraction of the disease. Spasm was exceedingly slight and rare, and collapse totally unknown.

When the disease recurred as Erythematic Enteritis, vomiting ceased to accompany it. Severe griping pains, urging to sudden, fluid, feculent evacuations, attacked chiefly in the night, in the midst of profound sleep, and so went on, but with diminished frequency: in the intervals there remaining an obtuse pain, with tenderness and a full and flaccid state of the abdomen generally. With these were associated rigors, and other symptoms of a febrile character; but they were for the most part unimportant, appeared to depend on the principal affection of the intestinal canal, and with it declined and passed away.

In January of the present year, these affections were combined with a sore throat, in which the tongue and parts about the fauces became tumid, sometimes with ulceration and a vivid redness, and attended with a very remarkable and complete loss of voice. Throughout their whole course there has been no unfavorable termination.

From the beginning I had opportunities of observing that the attempt to check the disease by opiates, warm purgatives, &c. served but to procrastinate its termination. It was not this, however, that influenced my choice of the remedies which I immediately applied, but that I had employed them in similar affections, with advantageous results, for several years; and if they were adapted to those I had before seen, they promised, as it seemed to me, to be still more efficacious in such as have been now described.

These remedies consisted of the tartarized antimony, with diluents and the neutral salts, sustaining the cure to the end by the strictest antiphlogistic regimen. Four grains of tartarized antimony were dissolved in eight ounces of water, and a quarter of this solution given every half-hour until the whole of it was taken; the person drinking freely, after the effect of the last portion, of lukewarm water. This, exhibited when nausea and vomiting were present, accelerated the evacuations, as well inferiorly as superiorly, and effected a rapid and perfect cure, which was, for the most part, completed in a few hours. But if, on some occasions, after a tranquil night, there remained a slight uneasiness in the belly on the following morning, shewing it to be still incomplete, the sulphate of soda or of magnesia, in the proportion of an ounce dissolved in a sufficient quantity of warm water, usually sufficed to remove this symptom entirely, and put an end to the disease.

In that variety in which the stomach has displayed less of irritability, the tartarized antimony has been omitted, and the neutral salts, then not likely to be rejected, alone entrusted with the cure, which they have effected always with remarkable certainty and quickness. They have been exhibited in the same manner and proportion as before mentioned; but where it has been thought useful to repeat them, this has been done sometimes by giving the full proportion at once, sometimes by dividing it into four parts, one of which is administered every hour, with perhaps little difference of effect; the leaning, however, in my own mind, being at present for the full proportion at once.

It has sometimes happened that the neutral salts have, by exerting some direct influence on the disease, seemed, instead of increasing the intestinal evacuations, to repress them altogether.

It has rarely occurred that any remains of the febrile state has lingered after the disappearance of the principal affection; but when, from the state of the tongue or otherwise, this has been suspected, the old and excellent fever powder, composed of the nitrate of potass, in the proportion of ten grains, with one eighth of a grain of tartarized antimony, repeated every three hours, has been usefully administered. In the affections of the throat, the dilute muriatic acid has, besides the usual treatment, been gargled, and also swallowed with beneficial effects.

By these means are accumulated or irritating matters removed from the intestines, or their offensive tendency corrected; the extensive mucous surfaces cleansed and soothed, and its disordered state replaced by a healthy condition; the febrile tendency repressed, and the sufferer restored to health.

In giving this account, it will be understood that I have considered the affections treated of as being indigenous, and totally unconnected with the fatal disease which, by the unwise abrogation of the laws directed to the exclusion of pestilence, has been introduced into the country. But, although I feel constrained to hold this opinion, I remarked with surprise the coincidence of this epidemic with the presence of the malignant form of the disease in other parts of Europe long before it made its appearance here; and I hoped, by communicating the treatment I had employed in the one, it might, if any similarity was found between them, be tried in the other. The mortality attendant on the methods of treatment hitherto employed has but too clearly shewn their futility. The frictions I have seen employed have appeared to me frivolous and useless, and taking the attention from more important objects; and I must deprecate the refusal of mild drinks to the craving sufferer, as unnecessary, cruel, and injurious.

*May 14th*, 1832.

